# Use of Point-of-Care Ultrasound to Confirm and Manage a Morel-Lavallée Lesion in the Emergency Department: A Case Report

**DOI:** 10.5811/cpcem.56952

**Published:** 2026-10-30

**Authors:** Siddharth Shanbhag, Ami Kurzweil

**Affiliations:** Eisenhower Health, Department of Emergency Medicine, Rancho Mirage, California

**Keywords:** Morel-Lavalle lesion, point-of-care ultrasound, emergency medicine, ultrasound-guided aspiration, case report

## Abstract

**Introduction:**

Morel-Lavallée lesions are closed, soft tissue degloving injuries that occur when shearing forces separate the subcutaneous tissue from the underlying fascia. This creates a potential space for hemolymphatic fluid accumulation. Morel-Lavallée lesions are frequently under-reported and often misdiagnosed as hematomas or abscesses, leading to potential mismanagement. While magnetic resonance imaging (MRI) is considered the gold standard for diagnosis, point-of-care ultrasound (POCUS) has been shown to be an effective adjunct that can rapidly confirm sonographic features consistent with Morel-Lavallée lesions, support MRI-established findings, and guide procedural intervention in the emergency department (ED) setting.

**Case Report:**

We describe a 20-year-old woman presenting to the ED with persistent right-sided hip pain two months after a pedestrian–motor vehicle collision. Initially misdiagnosed as a hematoma, an outpatient follow-up MRI identified a large Morel-Lavallée lesion, with POCUS confirming subcutaneous anechoic fluid collection in the ED. Ultrasound-guided aspiration yielded 150 mL of serosanguinous fluid, with real-time imaging confirming decompression. Laboratory analysis demonstrated elevated red blood cell and nucleated cell counts consistent with Morel-Lavallée lesion.

**Conclusion:**

This case highlights the integrated diagnostic and therapeutic role of point-of-care ultrasound as an adjunct to MRI, confirming sonographic features of a Morel-Lavallée lesion, guiding aspiration, and providing real-time postprocedural assessment within a single ED encounter in a patient presenting two months after injury.

## INTRODUCTION

A Morel-Lavallée lesion is a closed soft tissue degloving injury that occurs when a shearing force separates the subcutaneous tissue from the underlying fascia.^6^ This leads to the potential formation of a space between the two layers, typically filled with hemolymphatic fluid.^6^ Morel-Lavallée lesions can present acutely or be delayed in onset, and they can often be misdiagnosed due to their nonspecific external signs, such as ecchymosis and swelling resembling hematoma or abscesses.^5^,^6^ As a result, they are frequently underdiagnosed or mismanaged in the acute trauma setting.^5^

Early recognition is critical to guide management and prevent further complications such as skin necrosis and capsule formation.^6^ The gold standard for diagnosing Morel-Lavallée lesions is magnetic resonance imaging (MRI) due to its superior contrast and multiplanar capabilities, allowing for detection of capsule formation and lesion chronicity. However, point-of-care ultrasound (POCUS) has emerged as a rapid, cost-effective, radiation-free imaging modality within the emergency department (ED) that can serve as an effective adjunct to MRI, providing sonographic confirmation of characteristic features and guiding procedural intervention.^1^–^4^,^7^ In orthopedic and radiology practice, percutaneous ultrasound-guided aspiration has similarly been employed as a first-line management strategy for Morel-Lavallée lesions, with favorable outcomes reported across multiple patient series.^8^,^9^

The following case report demonstrates the utility of POCUS in the ED as an adjunct to confirm an MRI-established diagnosis of a Morel-Lavallée lesion in a patient who was initially misdiagnosed with a hematoma, and the subsequent use of POCUS for guided aspiration. While prior emergency medicine and orthopedic literature documents both POCUS-guided aspiration and ultrasound-guided drainage of these lesions,^1^–^4^,^7^–^9^ our report specifically illustrates POCUS used to confirm MRI findings, guide complete aspiration with real-time decompression monitoring, and facilitate definitive ED management.

## CASE REPORT

A 20-year-old woman presented to the ED with persistent right-sided hip and thigh pain. Two months prior to admission the patient was involved in a pedestrian–motor vehicle collision and was evaluated at a trauma center. Workup at that facility showed no fracture. Swelling over the hip was initially diagnosed as a hematoma. An MRI performed outpatient revealed evidence of a fluid collection suspicious for a Morel-Lavallée lesion measuring approximately 10 × 12 cm (Image 1). The patient was then lost to follow-up for 11 days. She was called by her primary physician and instructed to come into the ED.

Physical examination revealed swelling localized to the lateral aspect of the right hip, indurated skin, and boggy to palpation, with the affected leg being warm to touch with normal sensation. The patient’s vital signs were within normal limits, and sepsis screening did not indicate suspected infection. Point-of-care ultrasound of the right hip was performed by an attending emergency physician, revealing a large subcutaneous anechoic fluid collection measuring approximately 10 × 5 cm (Image 2). The collection contained internal echogenic debris but no sonographic evidence of abscess formation or swirl sign. These findings were consistent with the initial MRI diagnosis of Morel-Lavallée lesion, revealing a closed soft tissue degloving injury and supporting the clinical suspicion.

*CPC-EM Capsule*

What do we already know about this clinical entity?

*Morel-Lavallée lesions are closed degloving injuries that may mimic hematomas or abscesses and are frequently underdiagnosed.*

What makes this presentation of disease reportable?

*Point-of-care ultrasound confirmed a magnetic resonance imaging-diagnosed Morel-Lavallée lesion and enabled ultrasound-guided aspiration and real-time confirmation of decompression.*

What is the major learning point?

*Point-of-care ultrasound can confirm characteristic features of a Morel-Lavallée lesion, guide aspiration, and immediately assess procedural success.*

How might this improve emergency medicine practice?

*Point-of-care ultrasound can integrate bedside assessment and image-guided treatment of Morel-Lavallée lesions during a single emergency department encounter.*

Following ultrasound imaging, the patient underwent in-plane ultrasound-guided needle aspiration and drainage of the fluid collection over the right hip. Approximately 150 mL of serosanguinous fluid was aspirated. Subsequent real-time ultrasound imaging was performed to confirm complete aspiration and drainage of fluid that occupied the space between subcutaneous tissue and underlying fascia; imaging demonstrated a significant reduction of the aforementioned space (Image 3). The patient was provided a conservative management plan of using compression tape over the affected area, and she was instructed to follow up with general surgery for any reaccumulation or further management.

The fluid was sent for laboratory testing, specifically a body fluid culture, body fluid cell count, and body fluid differential count. The aspirated fluid appeared serosanguinous and amber in color. Laboratory analysis demonstrated a total fluid red blood cell count of 29,000/cm^2^ (reference range, ~1,000/ cm^2^) and a total nucleated cell count of 171/cm^2^ (0–5/cm^2^). These results were abnormal and consistent with degloving injury with a collection of hemolymphatic fluid, providing strong evidence of a Morel-Lavallée lesion.

## DISCUSSION

Morel-Lavallée lesions are closed soft tissue degloving injuries that result from shearing forces separating the hypodermis from the underlying fascia, creating a space for potential hemolymphatic fluid accumulation. These lesions remain underdiagnosed, in part because their clinical presentation frequently mimics more common conditions such as hematomas, seromas, or abscesses. The variable timing of symptom onset, which can range from weeks to even months and years, adds to diagnostic difficulty.^5^ Without recognition and proper management, Morel-Lavallée lesions can progress to chronic encapsulation, recurrent fluid collections, infection, impaired wound healing, and devascularization of surrounding tissue that could lead to necrosis.^6^

Magnetic resonance imaging is often considered the imaging gold standard for confirming and characterizing these lesions because it can evaluate capsule formation and lesion chronicity.^6^ However, MRI may not be readily available in the acute care setting due to cost, resource limitations, and scheduling delays. Ultrasound offers a rapid and cost-effective modality that can be performed at the bedside in the ED.^6^,^7^ Sonographic features, such as fusiform hypoechoic or anechoic fluid collections between the subcutaneous tissue and fascia sometimes with internal septations or echogenic debris, are highly suggestive of Morel-Lavallée lesions.^6^

Our case joins an existing body of literature that discusses the use of POCUS in the context of the treatment and management of Morel-Lavallée lesions. This case underscores POCUS as a cost-effective modality that is used in conjunction with MRI to confirm diagnosis, guide aspiration, and confirm near-complete decompression of hemolymphatic fluid-filled space.^1^–^4^, ^7^

This case is unique in that POCUS serves as an effective adjunct to MRI diagnosis, providing fast bedside imaging to confirm the presence of characteristic subcutaneous fluid collection consistent with a Morel-Lavallée lesion. In addition, POCUS aided in ruling out signs of abscess formation and other potential pathologies, which was critical in determining appropriate management. It also served a therapeutic role by guiding real-time aspiration of serosanguinous fluid and verifying complete decompression of the fluid collection, providing real-time confirmation of procedural success.^4^ Although POCUS has been demonstrated in this case to be an expedient option for the identification of a Morel-Lavallée lesion, its use is highly dependent on the sonographer’s proficiency.^6^

In addition, this case has several limitations inherent to a single case report. Most notably, the original MRI images are inaccessible from outside hospital records, precluding direct side-by-side comparison of MRI and POCUS findings; the MRI diagnosis is corroborated through documented physician notes. Additionally, long-term follow-up data beyond the presented ED encounter are limited.

## CONCLUSION

This case highlights the use of POCUS as an effective adjunct for the diagnosis of a Morel-Lavallée lesion. Point-of-care ultrasound provided rapid confirmation, excluded abscess formation, and guided successful aspiration with immediate postprocedural assessment. It confirmed, in real time, significant evacuation of the previously fluid-filled pocket occupying the space between subcutaneous tissue and underlying fascia thereby reducing long-term complications. The ability to integrate diagnosis and treatment in a single encounter underscores ultrasound’s value as a practical, cost-effective tool that can expedite care in the ED, prevent complications, and improve outcomes in patients presenting with posttraumatic soft tissue swelling with underlying Morel-Lavallée lesions. In this case, POCUS helped to raise suspicion by supporting the initial MRI diagnosis and facilitated ultrasound-guided aspiration and immediate postprocedural assessment. Magnetic resonance imaging remains the gold standard for definitive diagnosis and characterization of Morel-Lavallée lesions, while POCUS is a valuable early imaging adjunct in the ED.

## Figures and Tables

**Image 1:**
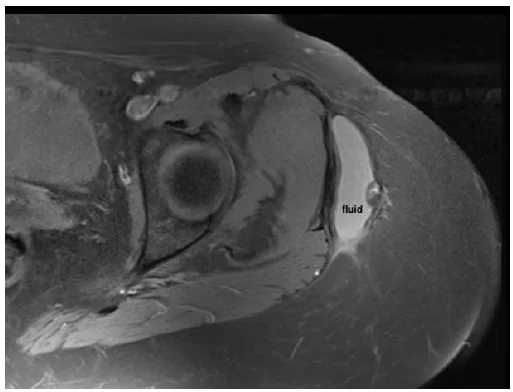
Representative case of an axial T2-weighted magnetic resonance imaging of a Morel-Lavallée lesion, demonstrating circumscribed thin-walled fluid collection at the interface between the subcutaneous fat and deep fascia, with fluid-fluid level and small globular focus of fat signal intensity. Image courtesy of Dr. J. Linklater, Radiopaedia.org, rID: 26390. Licensed under CC BY-NC-SA.^10^

**Image 2:**
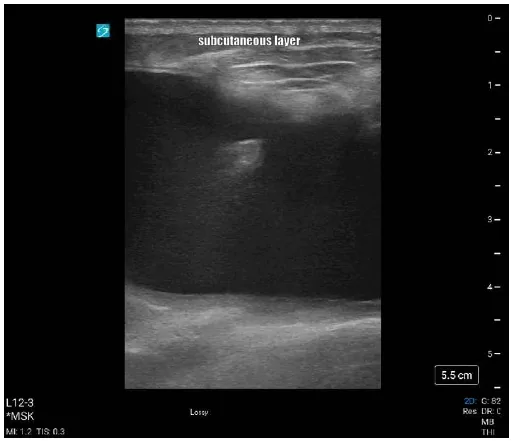
Point-of-care ultrasound revealing subcutaneous anechoic fluid collection measuring approximately 10 × 5 centimeters with evidence of hyperechoic debris.

**Image 3:**
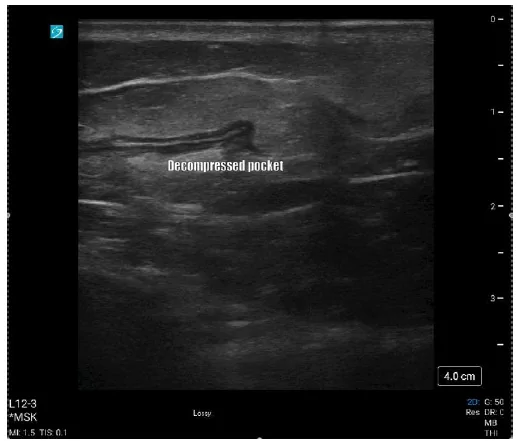
Postaspiration of the fluid collection over the right hip, demonstrating significant decompression between subcutaneous tissue and underlying fascia.

## References

[1] Gelber J, Sher W (2023). Morel-Lavallée Lesion diagnosed by point-of-care ultrasound: a case report and review of treatment strategies. J Emerg Med.

[2] Huang D, Killian M, Elbadri S (2023). Morel-Lavallée lesion of the distal thigh: emergency department diagnosis with point-of-care ultrasound. Radiol Case Rep.

[3] Koroshetz L, Berzins D, Lynch F (2024). Morel-Lavallée lesion diagnosed with point-of-care ultrasound. Ultrasound.

[4] Mack D, Miller J, Yuan F (2024). Diagnosis and treatment of a Morel-Lavallée lesion via point-of-care ultrasound. POCUS J.

[5] Joseph U, Thangayah JR (2023). What lies beneath: a case report of Morel-Lavallée Lesion. Proc Singapore Healthc.

[6] Diviti S, Gupta N, Hooda K (2017). Morel-Lavallée lesions: review of pathophysiology, clinical findings, imaging findings and management. J Clin Diagn Res.

[7] Johnson GM, Bernstein KM (2023). Noninvasive management of a large Morel-Lavallée lesion: a case report. Int J Sports Exerc Med.

[8] Martel Villagrán J, Díaz Candamio MJ, Bueno Horcajadas A (2018). Morel-Lavallée lesion: diagnosis and treatment with imaging techniques. Radiologia (Engl Ed).

[9] Leach SET, Wotherspoon M, King L (2020). Retrosacral Morel-Lavallée lesion: resolution with ultrasound-guided drainage and sclerotherapy. BJR Case Rep.

[10] Linklater J (2014). Morel-Lavallée lesion — left hip.

